# Luteolin inhibits respiratory syncytial virus replication by regulating the MiR-155/SOCS1/STAT1 signaling pathway

**DOI:** 10.1186/s12985-020-01451-6

**Published:** 2020-11-25

**Authors:** Saisai Wang, Yiting Ling, Yuanyuan Yao, Gang Zheng, Wenbin Chen

**Affiliations:** 1 Department of Colorectal Surgery, The First Affiliated Hospital, Zhejiang University School of Medicine, 79 Qingchun Road, Hangzhou, 310003 Zhejiang People’s Republic of China; 2 Department of Cardiology, The Second Affiliated Hospital, Zhejiang University School of Medicine, Hangzhou, 310009 Zhejiang People’s Republic of China

**Keywords:** Respiratory syncytial virus (RSV), Luteolin, Suppressor of cytokine signaling 1 (SOCS1), microRNA-155 (miR-155), Signal transducer and activator of transcription 1 (STAT1)

## Abstract

**Background:**

Respiratory syncytial virus (RSV) is a major cause of acute lower respiratory tract infection in infants, children, immunocompromised adults, and elderly individuals. Currently, there are few therapeutic options available to prevent RSV infection. The present study aimed to investigate the effects of luteolin on RSV replication and the related mechanisms.

**Material and methods:**

We pretreated cells and mice with luteolin before infection with RSV, the virus titer, expressions of RSV-F, interferon (IFN)-stimulated genes (ISGs), and production of IFN-α and IFN-β were determined by plaque assay, RT-qPCR, and ELISA, respectively. The activation of Janus kinase (JAK)-signal transducer and activator of transcription 1 (STAT1) signaling pathway was detected by Western blotting and luciferase assay. Proteins which negatively regulate STAT1 were determined by Western blotting. Then cells were transfected with suppressor of cytokine signaling 1 (SOCS1) plasmid and virus replication and ISGs expression were determined. Luciferase reporter assay and Western blotting were performed to detect the relationship between SOCS1 and miR-155.

**Results:**

Luteolin inhibited RSV replication, as shown by the decreased viral titer and RSV-F mRNA expression both in vitro and in vivo. The antiviral activity of luteolin was attributed to the enhanced phosphorylation of STAT1, resulting in the increased production of ISGs. Further study showed that SOCS1 was downregulated by luteolin and SOCS1 is a direct target of microRNA-155 (miR-155). Inhibition of miR-155 rescued luteolin-mediated SOCS1 downregulation, whereas upregulation of miR-155 enhanced the inhibitory effect of luteolin.

**Conclusion:**

Luteolin inhibits RSV replication by regulating the miR-155/SOCS1/STAT1 signaling pathway.

## Background

Human respiratory syncytial virus (RSV) is an enveloped, negative-sense, single-strand RNA (ssRNA) virus of the Pneumoviridae family [1]. RSV is the primary cause of respiratory infection in infants and children, resulting in pneumonia and bronchiolitis. Moreover, severe RSV infection is related to the development of recurrent wheezing or asthma [2]. In elderly individuals (65 years of age or older), RSV is also an important cause of respiratory infection [3]. Although continuous progress has been made in the development of an RSV vaccine [4], effective treatment against RSV is still urgently needed.

In natural infections, RSV replicates primarily in the airway epithelium. RSV infection or uptake by airway epithelial cells induces a direct antiviral response through the production of cytokines and chemokines. A549 cells (a human pulmonary alveolar cell carcinoma cell line with epithelial type II cell properties) are often used in RSV research. Type I interferons (IFNs), including IFN-α and IFN-β, are rapidly induced during viral infection and play a central role in restricting viral replication through the induction of a variety of antiviral effectors [5].

**Fig. 1:**
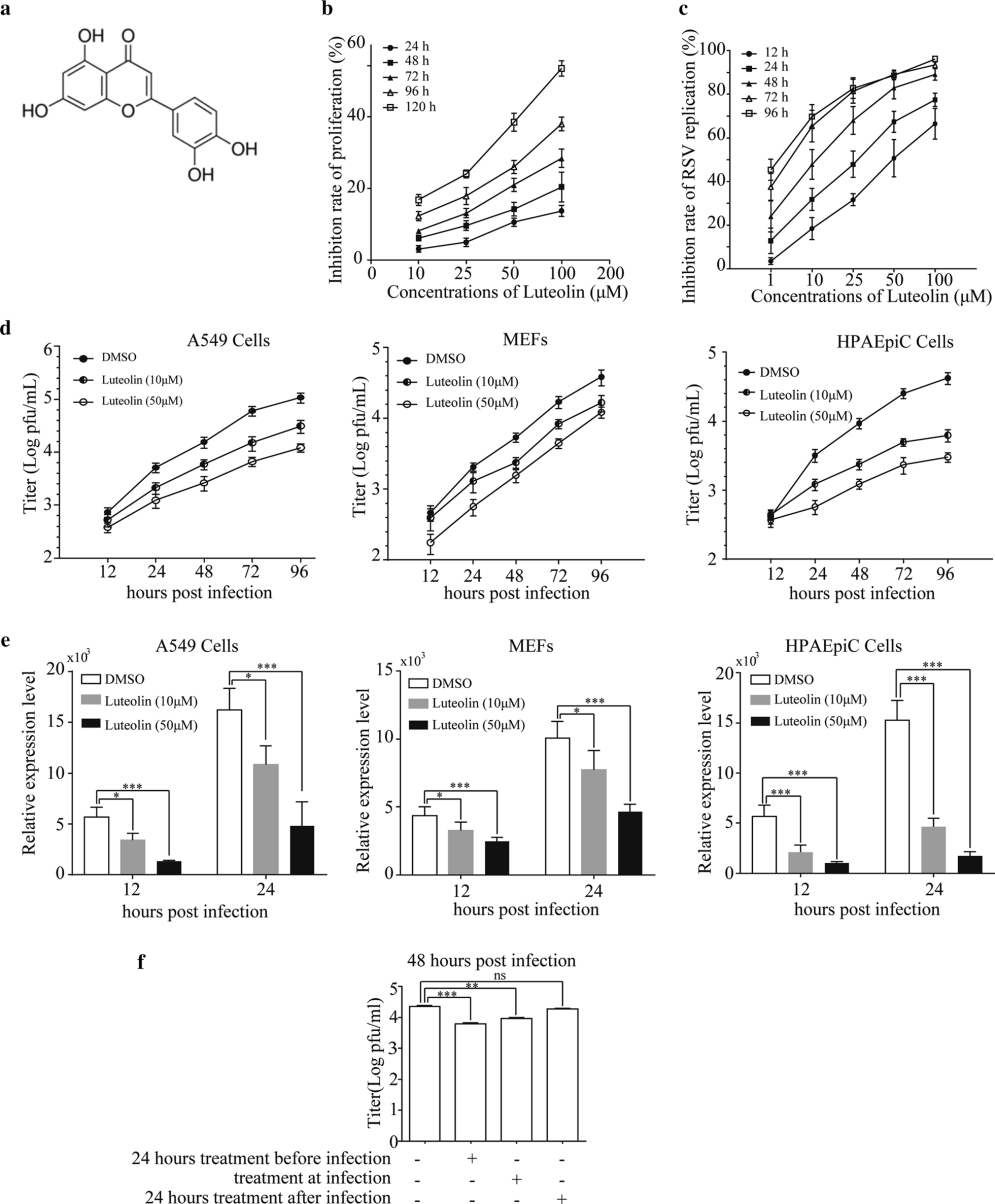
Luteolin inhibits RSV replication in A549 cells and MEFs. **a** Chemical structure of luteolin. **b** A549 cells were treated with the indicated concentrations of luteolin for 24, 48, 72, 96 and 120 h. Cell proliferation was measured by CCK-8 assay. **c** A549 cells were pre-treated with the indicated concentrations of luteolin for 24 h before infected with RSV at MOI = 0.05. After infection for 12, 24, 48, 72 and 96 h, cell culture medium was collected for plaque assay to determine the virus titer. A549 cells, MEFs and HPAEpiC cells were pre-treated with the indicated concentrations of luteolin for 24 h before infected with RSV at MOI = 0.05. At the indicated hours post infection, **d** virus titer was determined by plaque assay and **e** RSV-F mRNA expression was determined by RT-qPCR. **f** A549 cells were pre-treated or treated simultaneously with RSV infection or treated after RSV infection at MOI = 0.05. 48 h post infection, cell culture medium was collected for plaque assay to determine the virus titer. Data shown are means ± SEM. Statistical significance was examined by Students’ *t*-test. *P* < 0.05 was considered statistically significant. ^*^P < 0.05, ^**^P < 0.01, ^***^P < 0.001. *RSV* respiratory syncytial virus,; *CCK-8* Cell Counting Kit-8, *MOI* multiplicity of infection

Luteolin (Fig. 1a) is a common bioflavonoid found in a variety of fruits and vegetables. Luteolin has been reported to possess numerous beneficial medicinal properties, such as its anti-tumor, anti-inflammatory, cardioprotective, and neuroprotective effects [6–9]. Luteolin has been reported to exert inhibitory effects on dengue virus, Epstein-Barr virus (EBV), Japanese encephalitis virus, hepatitis B virus, and hepatitis C virus [10–14]. The mechanisms of these inhibitory effects include activation of extracellular signal-regulated kinase, downregulation of hepatocyte nuclear factor 4, inhibition of the host proprotein convertase furin, and repression of the EBV-induced promotion of immediate-early genes. Liu et al. reported that luteolin does not affect the replication of pseudorabies virus (PRV) in RAW264.7 cells but inhibits the expression of JAK, signal transducer and activator of transcription 1 (STAT1), STAT3, pSTAT1 and pSTAT3 [15]. Due to these properties, interest in the role of luteolin in RSV infection has increased.

MicroRNAs (miRNAs) are a class of small noncoding RNAs of ∼22 nt in length that usually exert their effects through directly binding to the 3′-untranslated regions (UTRs) of their target mRNAs [16], resulting in the degradation of these mRNAs or their translational inhibition [17, 18]. MicroRNA-155 (miRNA-155), a tumor-promoting miRNA, is processed from the B cell integration cluster. Studies have reported that miR-155 directly targets SHIP1, WEE1, VHL, TP53INP1, PU.1, BCL2, BCL6, SOCS1, and SOX family genes [19–21].

In this study, we demonstrated that luteolin has novel antiviral effect on RSV replication by inducing the expression of miR-155, which directly targets SOCS1, a negative regulator of STAT1 [22], leading to the upregulation of STAT1 phosphorylation and IFN-stimulated genes (ISGs) expression.

## Materials and methods

### Reagents

Luteolin (≥ 98%, HPLC) was purchased from Sigma-Aldrich (Shanghai, China) and dissolved in DMSO as a stock solution. Enzyme-linked immunosorbent assay (ELISA) kits for human and mouse IFN-α and IFN-β were purchased from R&D Systems (Minneapolis, MN). Antibodies against p-STAT1^Tyr701^, STAT1, p-JAK1^(Tyr1034/1035)^, JAK1, SOCS1, SOCS2, SOCS3, SOCS6, SHP-1, SHP-2 were purchased from Cell Signaling Technology (Danvers, MA, USA). Antibodies against CIS and GAPDH were purchased from Santa Cruz Biotechnology (Santa Cruz, CA). A Cell Counting Kit-8 (CCK-8) and horseradish peroxidase-conjugated goat anti-rabbit antibody were purchased from Beyotime Institute of Biotechnology (Haimen, China).

### Cell culture and treatment

HEp-2 and A549 cells were obtained from American Type Culture Collection (ATCC) and cultured in DMEM supplemented with 10% fetal bovine serum (FBS), 100 U/mL penicillin, and 100 μg/mL streptomycin at 37℃ in a humidified atmosphere of 5% CO_2_. Human pulmonary alveolar epithelial cells (HPAEpiC) were purchased from SciencCell (Shanghai, China) and cultured in complete alveolar epithelial cell medium (AEpiCM). Murine embryonic fibroblasts (MEFs) were obtained from C57BL/6 mouse embryos (14 days) and cultured in complete DMEM. AEpiCM was purchased from ScienCell and all the other culture reagents were purchased from Gibco (Shanghai, China). Cells were pretreated with vehicle or luteolin at the indicated concentrations for 24 h before infected with RSV at a multiplicity of infection (MOI) of 0.05. At different hours post infection (hpi), the whole cell culture was collected for plaque assay, supernatants were collected for ELISAs, or the cells were collected for RNA or protein extraction.

### Virus

The long strain of RSV was obtained from ATCC and propagated in HEp-2 cells. Briefly, the virus was added to a monolayer of HEp-2 cells and allowed to absorb for 2 h with rocking every 15 min. After 2 h of absorption, the medium was replaced with DMEM containing 2% FBS. Cells were left to grow for another 4–5 days until 80–90% cytopathic effects (CPE) were observed, and then the entire cell culture was collected. After three freeze–thaw cycles, the viruses were collected by centrifugation at 50,000 g for 1.5 h at 4 °C, and the remaining pellet was resuspended, aliquoted and stored at -80 °C before use. Plaque forming units (PFU) were determined by plaque assays with HEp-2 cells.

To determine the EC_50_ (concentration for 50% of maximal effect) value of luteolin against RSV infection, A549 cells were pretreated with various concentrations of luteolin before infected with RSV. After infection for 12, 24, 48, 72 and 96 h, the culture medium was collected for plaque assays to determine the viral titer. The viral replication inhibition rate was expressed as the percentage of viral replication compared to that in the DMSO-treated cells, and the EC_50_ was calculated. The selectivity indexes (SIs) for the various time points were calculated with the following equation: SI = CC_50_/EC_50_. The CC_50_ value (concentration of drug required to reduce cell viability by 50%) of luteolin in A549 cells was determined by CCK-8 assay.

### Plaque assay

Viral titer was determined by the plaque assay. HEp-2 cells were plated into 12-well plates and allowed to grow into a monolayer. Virus stocks, cell-free medium or lung homogenates from the mice were serially diluted tenfold at a volume of 200 μL and then incubated with HEp-2 cells. After 1-h incubation, the supernatants were removed, and the cells were overlaid with 1 mL of 1% (wt/vol) methylcellulose containing 50% (vol/vol) DMEM and 2% (vol/vol) FBS and cultured for another 5 days before the overlay medium was removed. Cells were fixed and stained with 2% (wt/vol) crystal violet in 20% (vol/vol) ethanol. The plaques were observed, and plaques in wells containing 30–100 plaques were counted. Viral titers were calculated using the following formula: viral titer (PFU/mL) = plaques × dilution × 5.

### CCK-8 assay

The CC_50_ value of luteolin in A549 cells was determined by the CCK-8 assay. A549 cells were plated into 96-well plates at a density of 1 × 10^3^/well. Twenty-four hours later, the culture media were replaced with media containing various concentrations of luteolin. After culturing for 24, 48, 72, 96 and 120 h, 10 μL of CCK-8 solution (Beyotime Institute of Biotechnology, Haimen, China) was added, and the cells were cultured for another hour before absorbance (Ab.) at 490 nm was measured using an ELISA microplate reader (EL800, BioTek Instruments, Winooski, VT, USA). The cell inhibition rate (I%) was calculated using the following equation: I% = (Ab. control − Ab. treated)/Ab. control × 100%. The CC_50_ value was determined.

### mRNA and miRNA quantification by reverse transcription-quantitative polymerase chain reaction (RT-qPCR) analysis

Total cellular RNA was extracted from cultured cells or lung homogenates with TRIzol reagent (Invitrogen). To analyze mRNA expression, 1 μg of RNA was reverse transcribed to cDNA with a PrimeScript RT Reagent Kit (TaKaRa, Beijing, China), and quantitative real-time PCR was then performed with SYBR Green qPCR Master Mix (TaKaRa). Sequences of the primers used for RT-qPCR are listed in Table 1. The calculated threshold cycle was normalized based on the value of β-actin amplified from the same cDNA, and the fold-change in expression was calculated as referenced to expression of the control.

**Table 1:** Primer sequences used in the experiment

Gene	Forward	Reverse
RSV-F	TTGGATCTGCAATCGCCA	CTTTTGATCTTGTTCACTTCTCCTTCT
hMX1	GTTTCCGAAGTGGACATCGCA	CTGCACAGGTTGTTCTCAGC
hOAS1	TGTCCAAGGTGGTAAAGGGTG	CCGGCGATTTAACTGATCCTG
hISG15	CGCAGATCACCCAGAAGATCG	TTCGTCGCATTTGTCCACCA
hIFN-α	GCCTCGCCCTTTGCTTTACT	CTGTGGGTCTCAGGGAGATCA
hIFN-β	CATTACCTGAAGGCCAAGGA	CAATTGTCCAGTCCCAGAGG
hβ-actin	CATGTACGTTGCTATCCAGGC	CTCCTTAATGTCACGCACGAT
mMX1	GACCATAGGGGTCTTGACCAA	AGACTTGTCTTTCTGAAAAGCC
mOAS1b	GAGGTCCACAGTTTAAGGAGTCC	GGTACGCCCACTGATGAGATT
mISG15	GGTGTCCGTGACTAACTCCAT	CTGTACCACTAGCATCACTGTG
mIFN-α4	TACTCAGCAGACCTTGAACCT	CAGTCTTGGCAGCAAGTTGAC
mIFN-β	ATGAGTGGTGGTTGCAGGC	TGACCTTTCAAATGCAGTAGATTCA
mβ-actin	GTATCCTGACCCTGAAGTACC	TGAAGGTCTCAAACATGATCT

To analyze miRNA levels, total RNA was reverse transcribed into cDNA with the miRcute miRNA First-Stand cDNA Synthesis Kit (Tiangen Biotech Co., Ltd., Beijing, China). The miRcute miRNA qPCR Detection Kit (SYBR Green; Tiangen Biotech Co., Ltd.) was used for qPCR. U6 was served as the housekeeping gene. The specific primers for miR-155 and U6 were synthesized by GenePharma (Shanghai, China). The primer sequences were as follows: miR-155, forward 5′-CTCAACTGGTGTCGTGGAGTCGGCAATTCAGTTGAGACCCCTAT-3′, reverse 5′-ACACTCCAGCTGGGTTAATGCTAATCGTGAT-3′; and U6, forward 5′-CTCGCTTCGGCAGCACA-3′, reverse, 5′-AACGCTTCACGAATTTGCG T-3′. All reactions were performed in triplicate, and the data were analyzed with the delta delta cycle threshold (CT) method of relative quantification.

### ELISA

To assess the production of cytokines, cell supernatants and bronchial alveolar lavage fluid (BALF) were collected. IFN-α and IFN-β levels were determined with commercial ELISA kits according to the manufacturer's instructions. The absorbance at 490 nm was read on an ELISA plate reader.

### Mouse infection

Female BALB/c mice (5–6 weeks old) were obtained from the Shanghai Laboratory Animal Company (SLAC; Shanghai, China). Current study received ethical approval from the Animal Care and Use Committee of Zhejiang University, and experiments and animal care were performed according to the approved protocols. Mice were randomly divided into three groups as follows: (i) mock-infected: mice were mock infected (DMEM only); (ii) RSV + PBS: mice were intraperitoneally injected with PBS 24 h before RSV infection; and (iii) RSV + luteolin: mice were intraperitoneally injected with 50 mg/kg luteolin 24 h before RSV infection. For RSV infection, mice were anesthetized and then intranasally inoculated with 5 × 10^5^ or 1 × 10^7^ PFU RSV in a total volume of 20 μL. At 1 or 3 days postinfection (dpi), the mice were euthanized. BALFs were harvested, and the lungs were collected for RNA extraction or fixed for hematoxylin and eosin (H&E) staining.

### Western blotting

Western blotting was performed using the standard SDS-PAGE separation technique. The harvested cells were washed with ice-cold PBS twice and lysed in 1 × RIPA buffer (Cell Signaling Technology, Danvers, MA) supplemented with 1 mM phenyl methyl sulfonyl fluoride (Solarbio, Beijing, China) and protease inhibitor cocktail. Protein concentrations were determined, and equal amounts of protein from each sample were separated by 10% SDS-PAGE and blotted onto PVDF membranes (Millipore, Billerica, MA, USA). The membranes were incubated overnight at 4 °C with antibodies against p-STAT1, total STAT1, p-JAK1, total JAK1, SOCS1, SOCS2, SOCS3, SOCS6, CIS, SHP-1, SHP-2 and GAPDH. Subsequently, the membranes were incubated with a horseradish peroxidase-conjugated goat anti-rabbit antibody for 1.5 h at room temperature. Immunoreactive protein bands were detected using an Odyssey scanning system (LI-COR, USA), quantified by ImageJ and normalized to the corresponding amount of total protein.

### Luciferase reporter assay

For the IFN-stimulated response element (ISRE) luciferase assay, A549 cells in 96-well plates were transfected with plasmid (pISRE-TA-luc, Beyotime) harboring the firefly luciferase gene under control of the ISRE promoter (ISRE-luc) with or without cotransfection with SOCS1 expression plasmid (Ruijie, Shanghai, China) or a control plasmid (empty pcDNA3.1 vector) with Lipofectamine 2000 (Invitrogen, Carlsbad, CA). Twenty-four hours after transfection, cells were treated with luteolin or vehicle for 24 h before infection with RSV at MOI of 0.05. At the indicated hpi, cells were collected, and luciferase activities were determined with the Bright-Glo luciferase assay system (Promega Corp., Madison, WI).

The reporter constructs pcDNA-Luc Wt SOCS1 (containing the 3′-UTR of SOCS1 downstream of the luciferase gene), pcDNA-Luc Mut SOCS1 (containing a mutated target seed sequence) and Renilla vector (reference) were synthesized by GenePharma (Shanghai, China). Cells were plated in 24-well plates and then cotransfected with pcDNA-Luc Wt SOCS1 or pcDNA-Luc Mut SOCS1 and miR-155 mimic or miR-155 negative control and Renilla vector using Lipofectamine 2000. Luciferase activities were analyzed using the Dual-Luciferase® Assay system (Promega Corp., Madison, WI, USA). Relative luciferase activity was obtained by normalizing the Renilla luciferase activity to the firefly luciferase activity.

### Cell transfection

A549 cells were transfected with a SOCS1 expression plasmid or a control plasmid (empty pcDNA3.1 vector) (Ruisai Technology, Shanghai, China) with or without 50 nM hsa-miR-155 mimic or 100 nM hsa-miR-155 inhibitor (GenePharma, Shanghai, China) using Lipofectamine 2000 (Invitrogen, Shanghai, China) according to the manufacturer's instructions. Control samples were transfected with a miRNA mimic negative control (miR-155 NC). After transfection for 6 h, cells were washed with ice-cold PBS twice and then cultured for an additional 24 h. The transfection efficiency was assessed by RT-qPCR or Western blotting.

### Statistical analysis

All data were analyzed using SPSS 17.0 software (SPSS Inc., Chicago, IL, USA). Data are presented as the means ± SEM. Comparisons between two groups were performed using the Student’s *t*-test. *P* < 0.05 indicated significance.

## Results

### Luteolin inhibits RSV replication in A549, MEFs and HPAEpiC cells

To elucidate the effects of luteolin on RSV replication, we first detected the CC_50_ values of luteolin in A549 cells by CCK-8 assay. As shown in Fig. 1b and Table 2, the CC_50_ values of luteolin in A549 cells at 24, 48, 72, 96 and 120 h were 549.4, 339.4, 214.9, 143 and 79.45 μM, respectively. We also determined the EC_50_ value of luteolin against RSV in A549 cells. As shown in Fig. 1c and Table 3, the EC_50_ values of luteolin against RSV at 12, 24, 48, 72 and 96 h were 49.94, 24.71, 10.16, 3.552 and 2.075 μM, respectively. Since most of the studies have focused on the early innate immune response elicited in the RSV-infected cells, we then chose concentrations of 10 and 50 μM for the next experiments at which will inhibit 50% of RSV replication at 12 and 48 h. Cells viability at 24, 48, 72 and 96 h after 10 μM luteolin treatment were 96.9%, 93.9%,91.9% and 87.7%, respectively. Cells viability at 24, 48, 72 and 96 h after 50 μM luteolin treatment were 89.4%, 85.8%,79.0% and 74.0%, respectively. A549 cells, MEFs and HPAEpiC cells were pretreated with luteolin for 24 h before infection with RSV at MOI of 0.05. At the indicated hpi, viral titer or RSV-F mRNA expression was determined by plaque assay (Fig. 1d) or RT-qPCR (Fig. 1e), respectively. As presented, luteolin significantly inhibited RSV replication in a dose-dependent manner. We also checked the RSV replication with luteolin treatment at different times and results showed that luteolin pre-treatment or treatment simultaneously with the virus infection could inhibit the RSV replication (*P* < 0.05) (Fig. 1f). However, luteolin addition 24 h after RSV infection had no effects on virus replication since the difference was not significant, indicating that the preventive effect of luteolin was more obvious than therapeutic effect.

**Table 2:** CC_50_ values of luteolin in A549 cells

	24 h	48 h	72 h	96 h	120 h
CC_50_ (μM)	549.4	339.4	214.9	143	79.45

**Table 3:** EC_50_ values and selectivity index (SI) of luteolin against RSV infection in A549 cells

	12 h	24 h	48 h	72 h	96 h
EC_50_ (μM)	49.94	24.71	10.16	3.552	2.075
SI		22.23391	33.40551	60.50113	68.91566

### Luteolin upregulates ISGs expression but has no effects on IFN-α and IFN-β production

**Fig. 2:**
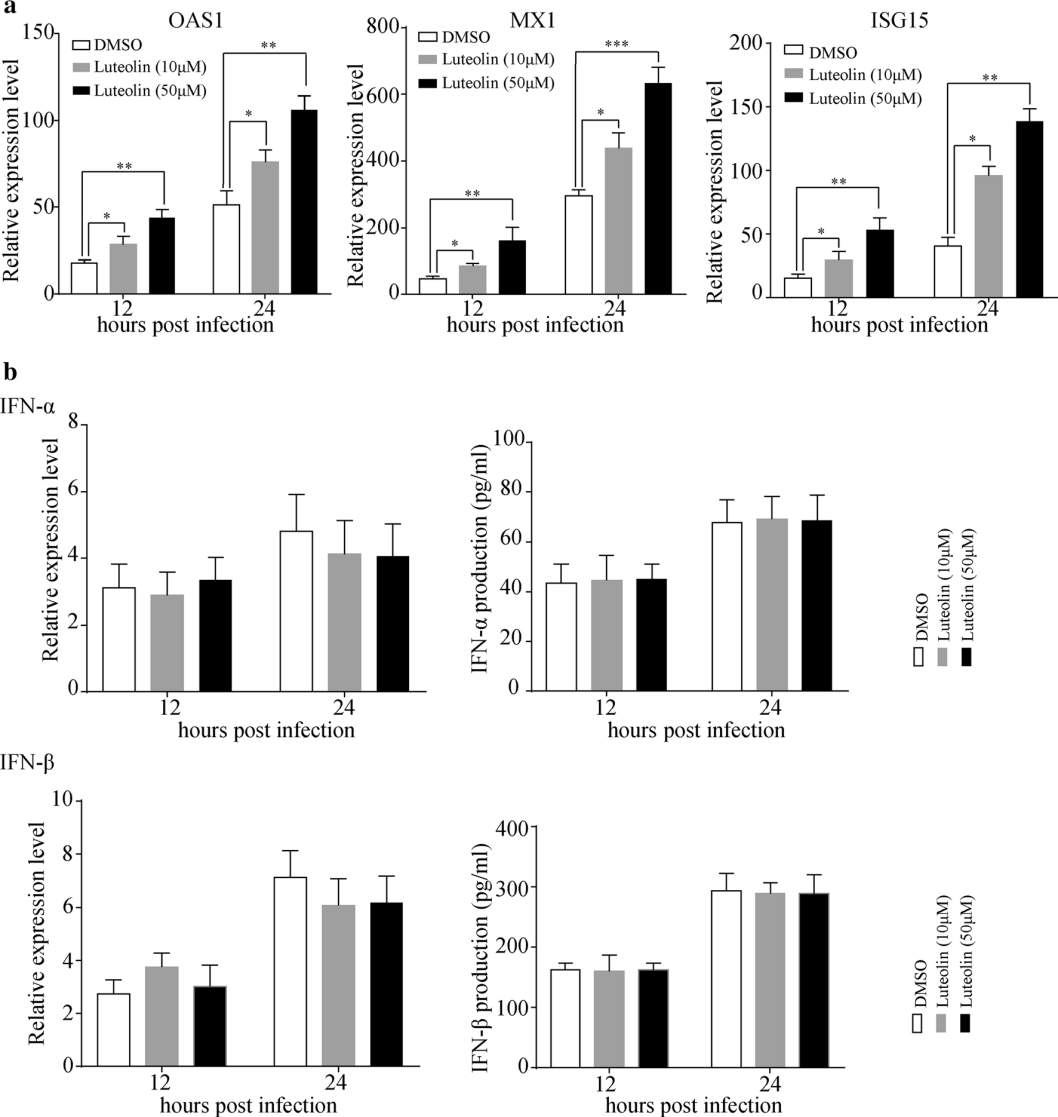
Luteolin increases MX1, OAS1 and ISG15 expressions in A549 cells. A549 cells were pre-treated with the indicated concentrations of luteolin for 24 h before infected with RSV at MOI = 0.05. At 12 or 24 h post infection, **a** RNA was extracted and RT-qPCR was performed to determine the MX1, OAS1 and ISG15 expressions. **b** IFN-α and IFN-β mRNA expressions or secretions in cell culture supernatants were determined by RT-qPCR or ELSIA, respectively. Data shown are means ± SEM. Statistical significance was examined by Students’ *t*-test. *P* < 0.05 was considered statistically significant. *P < 0.05, **P < 0.01, ***P < 0.001. *MX1* MX dynamin like GTPase 1, *OAS1* 2′-5′-oligoadenylate synthase 1, *ISG15* interferon stimulated gene 15, *RSV* respiratory syncytial virus, *MOI* multiplicity of infection

We next analyzed the expression of ISGs in A549 cells in the presence of RSV. Cells were pretreated with the indicated concentrations of luteolin, followed by RSV infection, and the mRNA expression levels of MX dynamin like GTPase 1 (MX1), 2′-5′-oligoadenylate synthase 1 (OAS1) and ISG15 were determined by RT-qPCR. Luteolin itself did not induce ISGs expressions (Additional file 1: Fig. 1). However, RSV infection increases ISGs expressions, and luteolin enhances RSV-induction of ISGs expressions (Fig. 2a). ISGs can be activated by type I IFN to execute their antiviral functions; thus, we detected IFN-α and IFN-β expression and secretion by RT-qPCR and ELISA, respectively. As shown in Fig. 2b, there were no large differences of IFN-α and IFN-β expressions and secretions with or without luteolin treatment, indicating that luteolin has no effect on type I IFN production.

### Luteolin inhibits RSV replication and alleviates RSV-triggered lung injury in mice

**Fig. 3:**
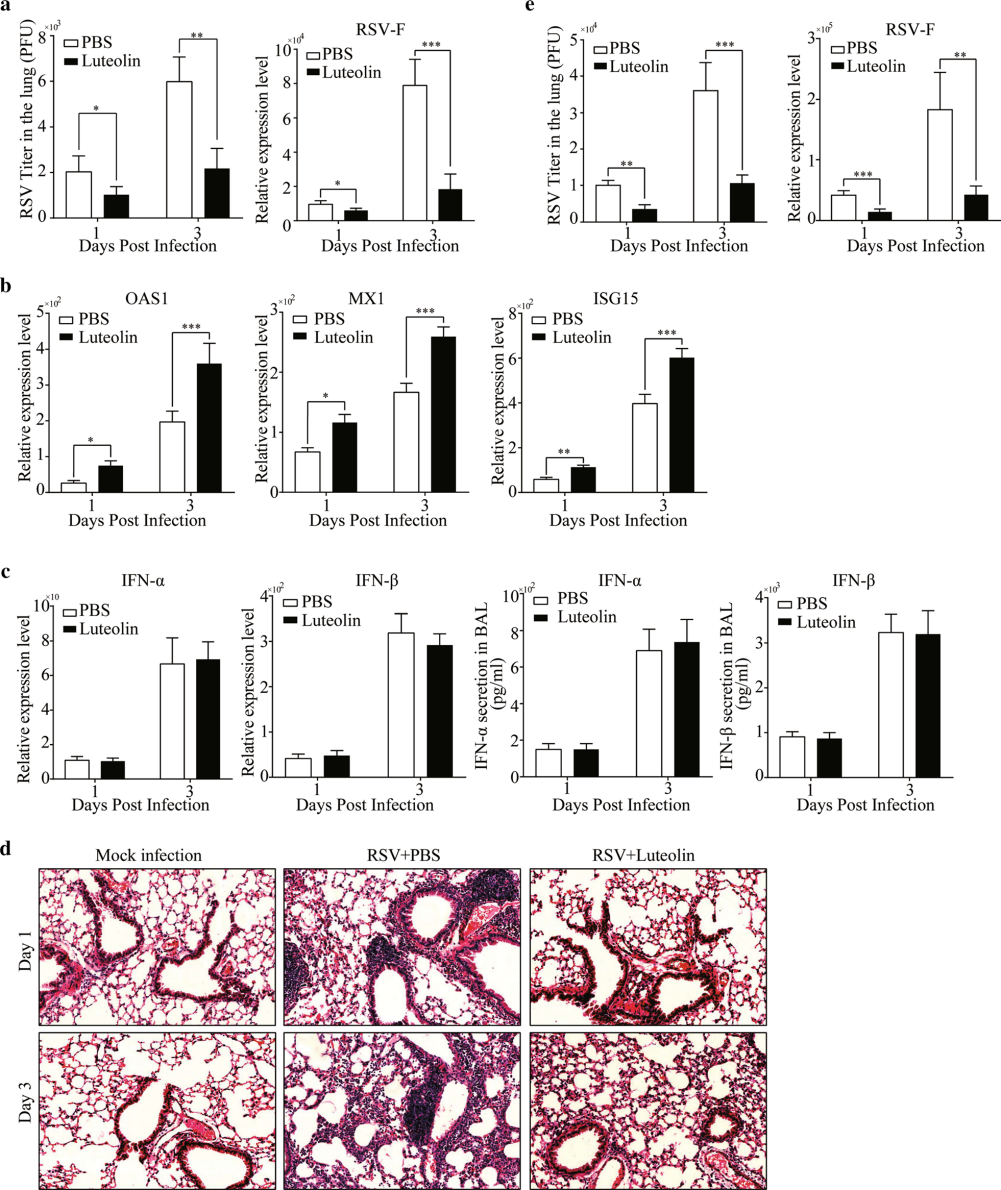
Luteolin inhibits RSV replication in vivo. BALB/c mice were intraperitoneally injected with luteolin before intranasally infected with 5 × 10^5^ PFU RSV. At 1- or 3-days post infection, **a** Virus titer and RSV-F mRNA expression in lung homogenates were measured by plaque assay and RT-qPCR, respectively. **b** The mRNA level of MX1, OAS1 and ISG15 in lung tissues were detected by RT-qPCR. **c** IFN-α and IFN-β mRNA expression in lung tissues or secretion in BALF were determined by RT-qPCR or ELISA, respectively. **d** Lung tissues were collected for H&E staining and the representative histological changes of the lung were presented (original magnification 200 × , Scale bar: 50 μm). **e** BALB/c mice were intraperitoneally injected with luteolin before intranasally infected with 1 × 10^7^ PFU RSV. At 1- or 3-days post infection, virus titer and RSV-F mRNA expression in lung homogenates were measured by plaque assay and RT-qPCR, respectively. Data shown are means ± SEM. Statistical significance was examined by Students’ *t*-test. *P* < 0.05 was considered statistically significant. *P < 0.05, **P < 0.01, ***P < 0.001. *RSV* respiratory syncytial virus, *MX1* MX dynamin like GTPase 1, *OAS1* 2′-5′-oligoadenylate synthase 1, *ISG15* interferon stimulated gene 15, *IFN* interferon, *BALF* bronchial alveolar lavage fluid

To characterize the effects of luteolin on RSV replication in vivo, viral replication, type I IFN production and the mouse inflammatory response were assessed following RSV infection. As predicted, we observed a decrease after luteolin treatment in viral titer (from 2034 ± 311 PFU/mL to 1018 ± 163 PFU/mL at 1 dpi and from 5980 ± 486 PFU/mL to 2180 ± 390 PFU/mL at 3 dpi) and RSV-F mRNA expression (Fig. 3a), accompanied by an increase in OAS1, MX1 and ISG15 expressions (Fig. 3b). RSV infection significantly induced IFN-α and IFN-β production in BALF, but there were no big differences in IFN-α and IFN-β levels between luteolin-treated and untreated mice (Fig. 3c). This finding was consistent with the in vitro results shown in Fig. 2b. Meanwhile, lung histopathology was evaluated by H&E staining. As shown in Fig. 3d, RSV infection caused severe pulmonary inflammation characterized by large lymphocytic infiltration and thickening of the alveolar wall, which were not observed in mock-infected mice. Mice in the luteolin-treated groups showed reduced histopathological changes as the lung had less lymphocytes infiltration. We also infected the mice with higher dosage of RSV (1 × 10^7^ PFU) and, excitingly, luteolin treatment significantly decreased the viral titer (from 10,120 ± 601 PFU/mL to 3600 ± 524 PFU/mL at 1 dpi and from 35,980 ± 3466 PFU/mL to 10,660 ± 1031 PFU/mL at 3 dpi) and RSV-F mRNA expression (Fig. 3e). Thus, we concluded that luteolin inhibits RSV replication both in vitro and in vivo.

### Luteolin upregulates ISGs through increased STAT1 phosphorylation

**Fig. 4:**
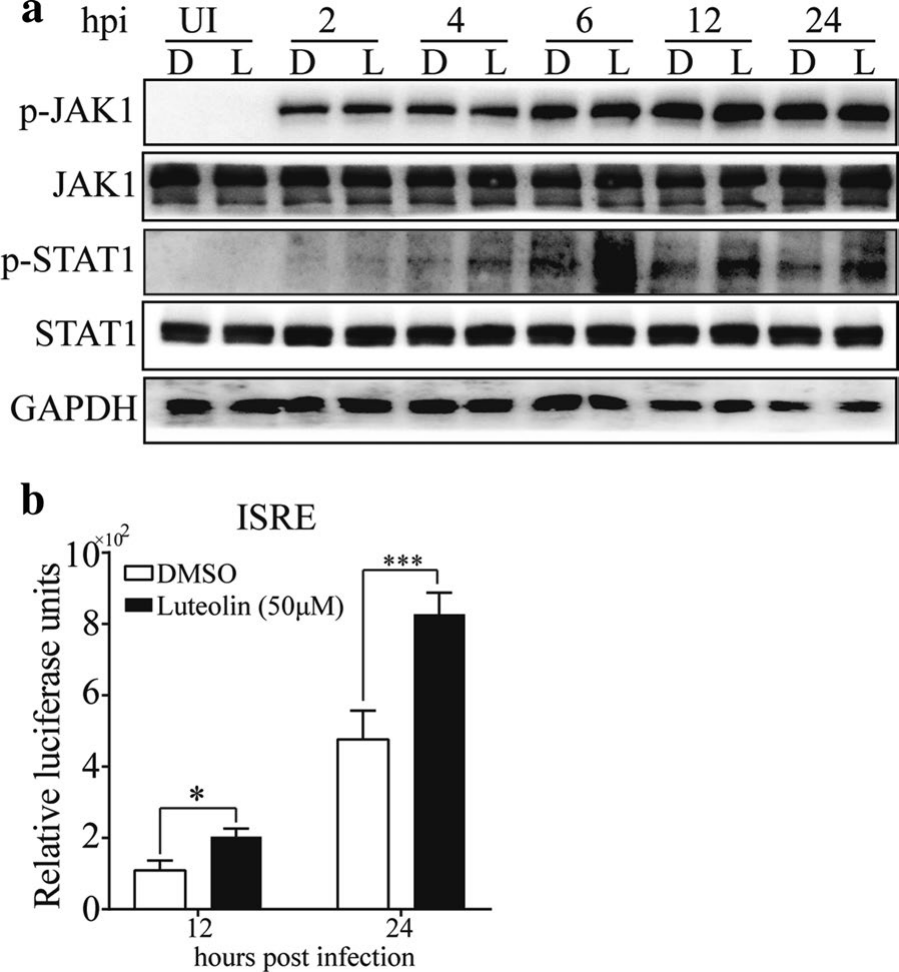
Luteolin enhances STAT1 phosphorylation and ISRE luciferase activity. **a** A549 cells were pre-treated with 50 μM luteolin for 24 h before infected with RSV at MOI = 0.05. At the indicated hours post infection, proteins were extracted for western blotting to detect the JAK, STAT1 phosphorylation. **b** A549 cells were transfected with pISRE-TA-luc for 24 h and cells were treated with luteolin for 24 h before infected with RSV. At 12 or 24 h post infection, cells were collected, and luciferase activities were determined. Data shown are means ± SEM. Statistical significance was examined by Students’ *t*-test. *P* < 0.05 was considered statistically significant. *P < 0.05, ***P < 0.001. *STAT1* signal transducers and activators of transcription 1, *MOI* multiplicity of infection, *JAK* Janus-activated kinase, *ISRE* interferon-stimulated response element, *RSV* respiratory syncytial virus

Since luteolin could upregulate ISGs expressions but not IFN-α/β production, we then determined whether this effect is mediated through STAT1. A549 cells were pretreated with luteolin or DMSO before infected with RSV. The expression levels of p-JAK1 and p-STAT1 were detected at different hpi. Interestingly, as shown in Fig. 4a, a significant increase in STAT1 phosphorylation was detected in luteolin-treated cells, and STAT1 phosphorylation peaked at 6 hpi. However, the level of phosphorylated JAK1 remained steady throughout stimulation at different hpi. We further examined ISRE promoter activity using a luciferase reporter assay and found that luteolin significantly enhanced ISRE activity (*P* < 0.05) (Fig. 4b). Taken together, these results suggested that luteolin upregulates ISGs through increased phosphorylation of STAT1. Thus, luteolin may positively regulate the type I IFN signaling pathway.

### Luteolin downregulates SOCS1 which negatively regulates STAT1 phosphorylation

**Fig. 5:**
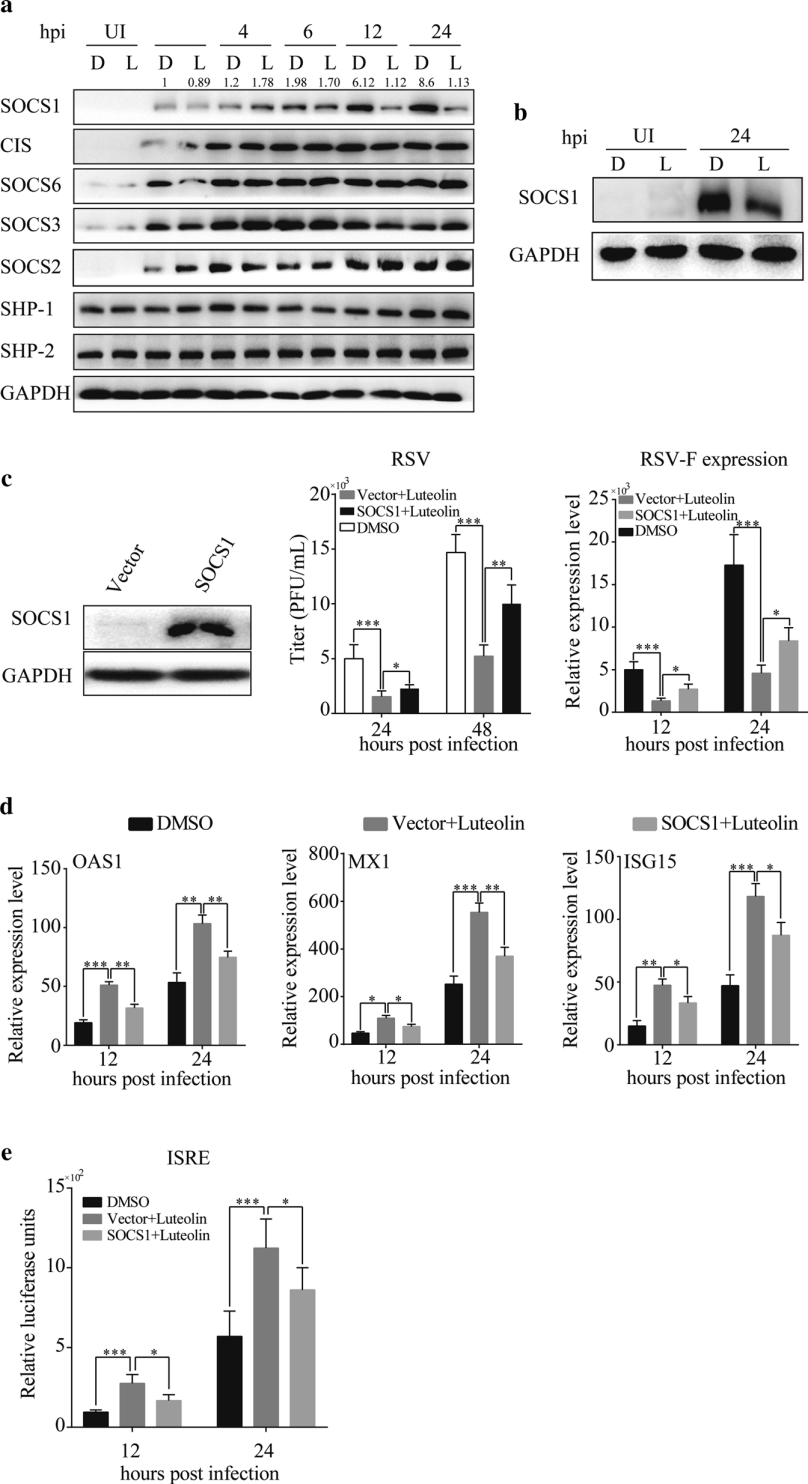
Over-expression of SOCS1 reverses the anti-viral effects of luteolin. **a** A549 cells were pre-treated with 50 μM luteolin for 24 h before infected with RSV at MOI = 0.05. At the indicated hours post infection, proteins were extracted for western blotting to detect the SOCS1, CIS, SOCS2, SOCS3, SOCS6, SHP-1 and SHP-2 expressions. **b** MEFs were pre-treated with 50 μM luteolin for 24 h before infected with RSV at MOI = 0.05. At 24 hpi, proteins were extracted for western blotting to detect the SOCS1 expression. A549 cells were transfected with SOCS1 plasmid or control plasmid. 24 h later, cells were stimulated with luteolin for 24 h before infected with RSV. **c** At the indicated hours post infection, virus titer or RSV-F mRNA expression was determined by plaque assay or RT-qPCR, respectively. **d** At the indicated hours post infection, cells were collected for RNA extraction and RT-qPCR was performed to detect the MX1, OAS1 and ISG15 expressions. **e** A549 cells were co-transfected with ISRE-luc and SOCS1 plasmid or control plasmid. 24 h later, cells were stimulated with luteolin for 24 h before infected with RSV. At 12 or 24 h post infection, cells were collected, and luciferase activities were determined. Data shown are means ± SEM. Statistical significance was examined by Students’ t-test. *P* < 0.05 was considered statistically significant. *P < 0.05, **P < 0.01, ***P < 0.001. *SOCS* suppressor of cytokine, *RSV* respiratory syncytial virus, *CIS* cytokine-induced STAT inhibitor, *SHP* protein-tyrosine phosphatase, *MOI* multiplicity of infection

To gain insight into the mechanisms by which luteolin modulates STAT1 signaling, the expression levels of SOCS1, SOCS2, SOCS3, SOCS6, CIS, SHP-1 and SHP-2, proteins which are known to negatively regulate STAT1, were analyzed. A decrease in SOCS1 expression was observed in luteolin-treated A549 cells and MEFs (Fig. 5a, b). Thus, we overexpressed SOCS1 in A549 cells to determine the effects of SOCS1 in this process. As shown in Fig. 5c, overexpression of SOCS1 partly reversed luteolin-induced viral inhibition of which the viral titer changed from 1542 ± 212 PFU/mL to 2267 ± 145 PFU/mL (*P* < 0.05) at 24 hpi and 5233 ± 419 PFU/mL to 10,017 ± 700 PFU/mL (*P* < 0.01) at 48 hpi, as well as RSV-F mRNA expression. We also detected MX1, OAS1 and ISG15 expressions by RT-qPCR (Fig. 5d) and ISRE promoter activity by luciferase assay (Fig. 5e). As expected, luteolin significantly decreased the ISGs expressions and ISRE activity, while SOCS1 rescued this decrease (*P* < 0.05). These results suggest that luteolin upregulates STAT1 phosphorylation by decreasing SOCS1 expression, thus increasing the expression of ISGs to inhibit RSV replication.

### Luteolin downregulates SOCS1 expression by inducing miR-155 expression

**Fig. 6:**
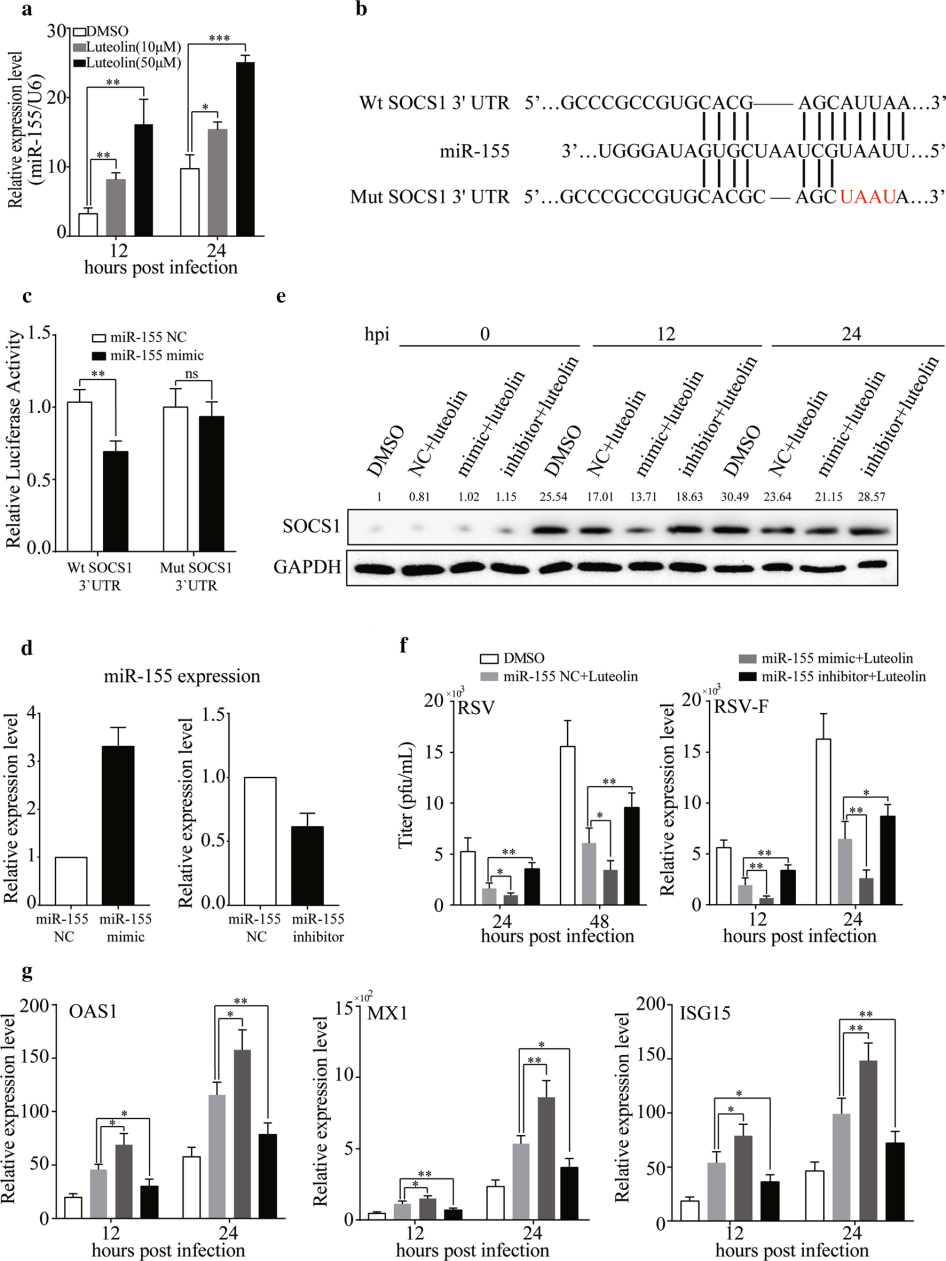
SOCS1 is a target of miR-155. **a** A549 cells were pre-treated with luteolin for 24 h before infected with RSV. At 12 or 24 h post infection, cells were collected for RT-qPCR to detect the miR-155 expression. **b** Putative miR-155 binding sites in the SOCS1 3′-UTR. The sites targeted by mutagenesis are indicated. **c** Dual luciferase reporter assay was performed in A549 cells that co-transfected with pcDNA-Luc Wt SOCS1 or pcDNA-Luc Mut SOCS1 and miR-155 mimic or miR-155 negative control to detect the luciferase activity at 48 h after transfection. Renilla luciferase activities normalized for firefly luciferase are presented. A549 cells were transfected with miR-155 mimc or miR-155 inhibitor for 24 h, **d** The mRNA expression of miR-155 was detected by RT-qPCR. **e** Cells were treated with luteolin for 24 h before infected with RSV and proteins were extracted at the indicated hours post infection to detect the SOCS1 expression by Western Blotting. **f** At the indicated hours post infection, virus titer or RSV-F mRNA expression were determined by plaque assay or RT-qPCR, respectively. **g** At the indicated hours post infection, cells were collected for RNA extraction and RT-qPCR was performed to detect the MX1, OAS1 and ISG15 expressions. Data shown are means ± SEM. Statistical significance was examined by Students’ t-test. *P* < 0.05 was considered statistically significant. *P < 0.05, **P < 0.01, ***P < 0.001. *SOCS* suppressor of cytokine, *RSV* respiratory syncytial virus, *MOI* multiplicity of infection, *STAT1* signal transducers and activators of transcription 1, *JAK* Janus-activated kinase

Previous studies reported that SOCS1 is a target of miR-155 [22, 23]; therefore, we speculated that miR-155 plays a key role in the mechanism by which luteolin attenuates SOCS1 expression in A549 cells. To evaluate this hypothesis, we first detected miR-155 expression in cells pretreated with luteolin by RT-qPCR (Fig. 6a). The expression level of miR-155 was significantly increased in the luteolin-stimulated groups compared with that in the control groups (*P* < 0.05). Then, we performed a luciferase reporter assay in A549 cells. As shown in Fig. 6b, c, miR-155 significantly inhibited the luciferase reporter activity of the Wt but not Mut SOCS1 3′-UTR, indicating that SOCS1 is a direct target of miR-155. Western blotting analysis of SOCS1 in miR-155 mimic- or miR-155 inhibitor-transfected cells confirmed that miR-155 downregulated the expression of SOCS1 in A549 cells infected with RSV (Fig. 6d, e).

Having demonstrated that SOCS1 is a direct target of miR-155, we further explored the effect of miR-155 on luteolin-induced viral inhibition. A549 cells were transfected with miR-155 mimic or miR-155 inhibitor before being treated with luteolin. Inhibition of miR-155 rescued the inhibitory effect of luteolin, whereas upregulation of miR-155 enhanced this inhibitory effect (Fig. 6f). These effects were tightly related to changes in ISG expression (Fig. 6g).

## Discussion

In the present study, the effects of luteolin on RSV replication were examined with the aim of determining the potential antiviral mechanism of luteolin. The significant observations of this study are as follows: (1) luteolin inhibits RSV replication both in vitro and in vivo; (2) luteolin upregulates MX1, OAS1 and ISG15 expression and STAT1 phosphorylation but has no significant effects on type I IFN production; (3) luteolin downregulates the expression of SOCS1, which is a negative regulator of STAT1; (4) luteolin increases the expression of miR-155, the direct target of which is SOCS1 in RSV-infected A549 cells; (5) miR-155 mimic enhances the antiviral effects of luteolin, whereas miR-155 inhibitor weakens these antiviral effects. Thus, we concluded that luteolin inhibits RSV replication by regulating the miR-155/SOCS1/STAT1 signaling pathway.

Studies have reported that luteolin exerts various effects via miRNA modulation. Luteolin can inhibit tumorigenesis and induce the apoptosis of non-small cell lung cancer cells, gastric cancer cells and esophageal cancer cells via upregulation of miR-34a [24–26]. Luteolin inhibits the proliferation and induces the apoptosis of prostate cancer cells by downregulating miR-301 [27]. Luteolin was also reported to inhibit ischemia/reperfusion-induced myocardial injury via downregulation of miRNA-208b-3p [28]. In our study, we also found that luteolin can inhibit A549 cells replication in a dose- and time-dependent manner (Fig. 1b). Thus, we speculated that the viral inhibition may be partly related to the cell’s death. As a result, the concentrations at 10 and 50 μM of luteolin were chosen for the following experiments at which the inhibition rates of cells proliferation were 12.27% and 26%, respectively at 96 h post stimulation. Consistently, miR-155 expression was upregulated in A549 cells after treatment with luteolin. However, luteolin has been found to decrease miR-155 expression in both gastric cancer and breast cancer cells [29, 30]. This finding is very intriguing and requires further study.

JAK/STAT1 activation results in the induction of various antiviral genes and the suppression of cytokine signaling (SOCS) genes, which form a negative feedback loop for IFN signaling [31]. RSV infection in A549 cells induces SOCS1 and SOCS3 expressions, which further reduces STAT phosphorylation and facilitates the virus survival [32]. In our study, we also observed the induced expressions of SOCS1 and SOCS3 (Fig. 5a). However, after luteolin treatment, SOCS1 was downregulated, decreasing the attenuation of STAT1 phosphorylation. This result implies that luteolin can enhance the antiviral effects of IFNα/β during RSV infection.

MiR-155 plays crucial roles in the immune response, tumorigenesis and stem cell differentiation [33–36] and has been reported to target and repress many genes including SOCS1, TNF-α, and SHIP1 [18–20]. Dudda et al*.* reported that miR-155 and its target gene, SOCS1, are key regulators of effector CD8( +) T cells as they affect cytokine signaling through STAT5 [37]. Wang et al*.* found that in acute pancreatitis, miR-155 targets SOCS1 to regulate the Th17 cell/Treg ratio, thus mediating disease severity [38]. MiR-155 was also shown to function as a pro-proliferative regulator during liver regeneration by facilitating the cell cycle and directly targeting SOCS1 [39]. In this study, we demonstrated that SOCS1 is the direct target of miR-155, which is consistent with the results of previous reports. Inhibition of miR-155 restored SOCS1 expression, and overexpression of miR-155 decreased SOCS1 expression.

## Conclusion

Taken together, the results of the current study demonstrated that luteolin can inhibit RSV replication both in vitro and in vivo through the induction of miR-155, which targets SOCS1, leading to enhanced activation of STAT1 phosphorylation and ISG expression. These results may facilitate the development of valuable therapeutic strategies to treat RSV infection by enhancing the STAT1 signaling pathway with traditional Chinese medicine or miRNAs.

## Supplementary information

**Additional file 1**. **Figure 1**: Luteolin cannot induce MX1, OAS1, ISG15, IFN-α, IFN-β expressions in A549 cells. A549 cells were treated with luteolin at 50 μM for 24 or 48 hours before RNA was extracted and RT-qPCR was performed to determine the MX1, OAS1, ISG1, IFN-α and IFN-β expressions.

## Data Availability

All data supporting the conclusions of this article are included in this published article.

## Abbreviations

RSV: Respiratory syncytial virus
ISG: Interferon-stimulated gene
JAK: Janus kinase
STAT1: Signal transducer and activator of transcription 1
SOCS1: Suppressor of cytokine signaling 1
miR-155: MicroRNA-155
IFN: Interferon
hpi: Hours post infection
PFU: Plaque forming units
dpi: Days post infection
