# Supplementary figures and images for: Luteolin inhibits respiratory syncytial virus replication by regulating the MiR-155/SOCS1/STAT1 signaling pathway
Source: Virol J. 2020 Nov 25;17:187. doi: 10.1186/s12985-020-01451-6 (PMC7688008; doi:10.1186/s12985-020-01451-6)

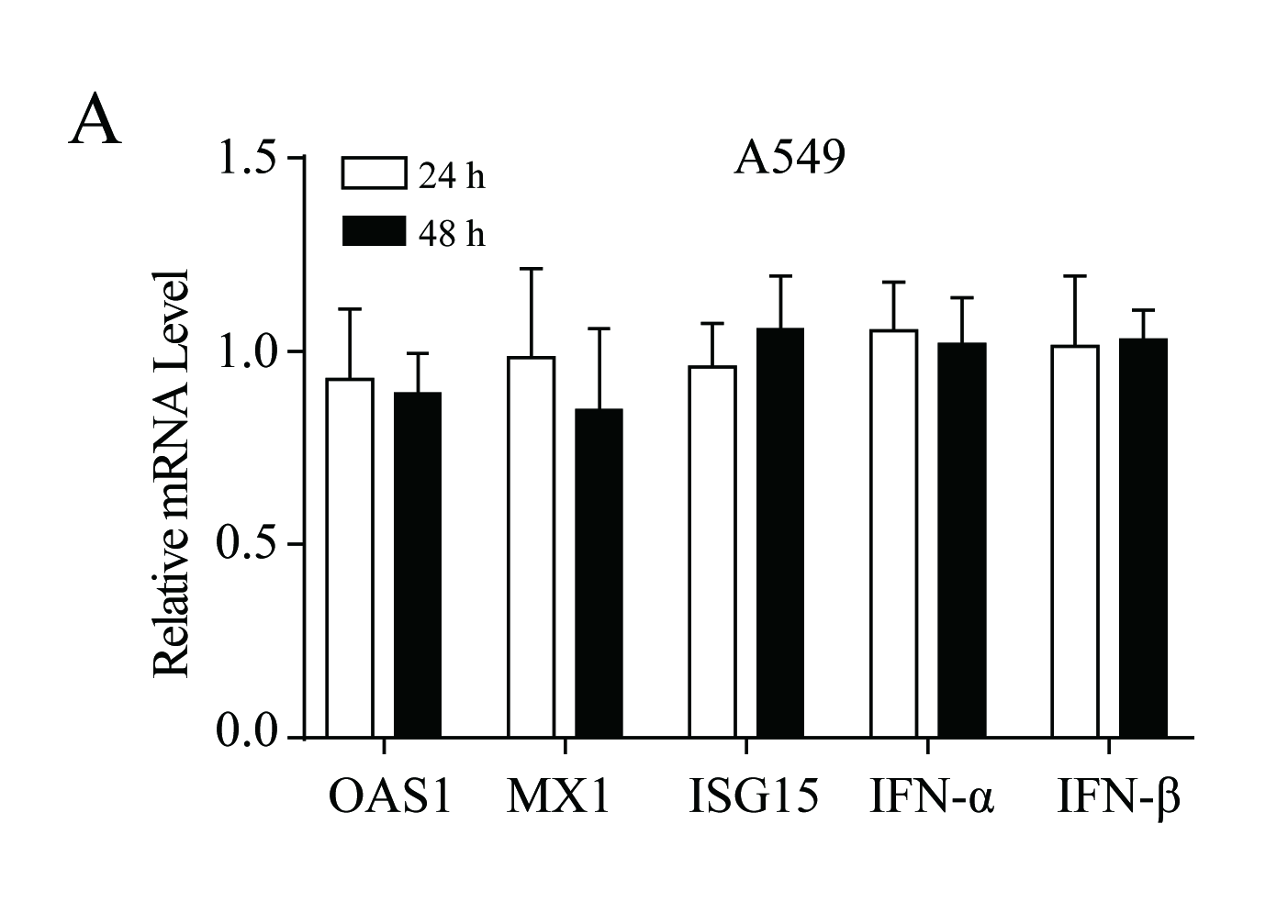

Supplement: Supplementary file 1 — Additional file 1. Figure 1: Luteolin cannot induce MX1, OAS1, ISG15, IFN-α, IFN-β expressions in A549 cells. A549 cells were treated with luteolin at 50 μM for 24 or 48 hours before RNA was extracted and RT-qPCR was performed to determine the MX1, OAS1, ISG1, IFN-α and IFN-β expressions. [file 12985_2020_1451_MOESM1_ESM.tif]
